# Advances in adrenal tumors 2018

**DOI:** 10.1530/ERC-18-0138

**Published:** 2018-07-28

**Authors:** J Crona, F Beuschlein, K Pacak, B Skogseid

**Affiliations:** 1 Department of Medical SciencesUppsala University, Uppsala, Sweden; 2 Medizinische Klinik und Poliklinik IVKlinikum der Universität München, Munich, Germany; 3 Klinik für EndokrinologieDiabetologie und Klinische Ernährung, UniversitätsSpital Zürich, Zürich, Switzerland; 4 Section on Medical NeuroendocrinologyEunice Kennedy Shriver National Institute of Child Health & Human Development, National Institutes of Health, Bethesda, Maryland, USA

**Keywords:** pheochromocytoma, paraganglioma, adrenocortical carcinoma, adrenocortical adenoma, adrenal tumor

## Abstract

This review aims to provide clinicians and researchers with a condensed update on the most important studies in the field during 2017. We present the academic output measured by active clinical trials and peer-reviewed published manuscripts. The most important and contributory manuscripts were summarized for each diagnostic entity, with a particular focus on manuscripts that describe translational research that have the potential to improve clinical care. Finally, we highlight the importance of collaborations in adrenal tumor research, which allowed for these recent advances and provide structures for future success in this scientific field.

## Introduction

Clinically unapparent adrenal tumors are found in 2–10% of the population worldwide. Cases that do require treatment are enriched in risk populations particularly those with hypertension or genetic risk factors (overview in Fassnacht *et al*. 2016, Young *et al*. 2017). Those that are associated with endocrine disturbances can cause severe patient morbidity and remains difficult to recognize and diagnose. Metastatic disease is a rare but lethal condition that can only be cured through complete surgical resection. Thus, in order to improve outcomes from adrenal tumor disease, there is a clear need for improved diagnostic methods, prognostic and predictive biomarkers but most importantly for improved therapeutic strategies.

Here, we performed a systematic review of research papers with an electronic publication date during 2017 and that were focused on adrenal tumors. A total of 349 papers including basic science and clinical studies were identified – 154 on pheochromocytomas (PCCs) and paragangliomas (PGLs, collectively denoted PPGLs), 177 on adrenocortical tumors and 18 that fell into a general adrenal tumor category. In this review, we have referenced 110 of these manuscripts and selected three prominent topics that we felt deserved special attention (Table 1). We also identified 21 active interventional studies on clinicaltrials.gov, 13 for PPGLs and 10 for adrenocortical tumors (Tables 2 and 3). Key drivers behind this research included researchers that were affiliated to the United States National Institutes of Health (NIH), European Network for the Study of Adrenal Tumors (ENSAT) or the newly formed American-Australian-Asian Adrenal Alliance (A5). In numbers, they were involved in 37% of the research papers assessed in our survey, with the number increasing to 50, 62, and 82% if considering papers with impact factor ≥3, ≥6, and ≥9, respectively. Two placebo-controlled clinical trials for adrenal tumors (FIRSTMAPP and ADIUVO) were both initiated and executed through members of ENSAT. NIH and A5 were both associated with six clinical trials each. Guidelines relevant to adrenal tumor patients are summarized in Table 4.

**Table 1:** Selected topics and studies during 2017.

Topic	Title	Reference
PPGL, molecular characterization and data repository	Comprehensive molecular characterization of pheochromocytoma and paraganglioma	Fishbein *et al*. (2017*b*)
**ACC, translating molecular characteristics into robust prognostic markers**	**DNA methylation is an independent prognostic marker of survival in adrenocortical cancer**	Jouinot *et al*. (2017)
	**Assessment of VAV2 expression refines prognostic prediction in adrenocortical carcinoma**	Sbiera *et al*. (2017)
APA, candidates for drug repositioning	Macrolides blunt aldosterone biosynthesis: a proof-of-concept study in KCNJ5 mutated adenoma cells *ex vivo*	Caroccia *et al*. (2017)
	Macrolides selectively inhibit mutant KCNJ5 potassium channels that cause aldosterone-producing adenoma	Scholl *et al*. (2017)

ACC, adrenocortical carcinoma; APA, aldosterone-producing adenoma; PPGL, pheochromocytoma and paraganglioma.

**Table 2:** Recruiting clinical trials for PPGL.

Intervention	Study design	Setting	Recruitment target, *n* patients	NCT number
**Randomized studies**				
Sunitinib	Phase II, placebo controlled	Palliative	74	Nbib1371201
Phenoxybenzamine vs doxazosin	Phase III	Curative and palliative	60	Nbib3176693
Phenoxybenzamine vs doxazosin	Phase IV	Curative	134	Nbib1379898
**Non-randomized studies**				
Cabozantinib	Phase II, single arm	Palliative	22	Nbib2302833
SGI-110 (Guadecitabine)	Phase II, single arm	Palliative	70	Nbib3165721
Lenvatinib	Phase II, single arm	Palliative	25	Nbib3008369
Lu-177-DOTATATE	Phase II, single arm	Palliative	90	Nbib3206060
**Studies investigating multiple disease**				
^131^I-MIBG	Phase II, single arm	Palliative	80	Nbib107289
^131^I-MIBG	Phase II, single arm	Palliative	100	Nbib1850888
Nivolumab and ipilimumab	Phase II, single arm	Palliative	707	Nbib2834013
ONC-201	Phase II, single arm	Palliative	24	Nbib3034200
Pembrolizumab	Phase II, single arm	Palliative	250	Nbib2721732
PEN-221	Phase I/IIa, single arm	Palliative	120	Nbib2936323

NCT, ClinicalTrials.gov registry number; PPGL, pheochromocytoma and paraganglioma.

**Table 3:** Recruiting clinical trials for adrenal cortical tumors.

Intervention	Design	Setting	Recruitment target, *n* patients	NCT number
**Randomized**				
Mitotane	Phase III	Adjuvant	200	Nbib777244
ATR-101	Phase II, placebo controlled	Palliative (symptom reduction)	16	Nbib3053271
Surgery + medical therapy vs medical therapy	Randomized study	Curative	110	Nbib2364089
**Non-randomized**				
RF-ablation	Single-arm study	Curative	25	Nbib2756754
Pembrolizumab	Phase II, single arm	Palliative	39	Nbib2673333
**Studies investigating multiple diseases**				
ABBV-176	Phase I, single arm, basket	Palliative	100	Nbib3145909
Cabozantinib-S-malate	Phase II, single arm	Palliative	110	Nbib2867592
Nivolumab and ipilimumab	Phase II, single arm	Palliative	57	Nbib3333616
Nivolumab and ipilimumab	Phase II, single arm	Palliative	707	Nbib2834013
Pembrolizumab	Phase II, single arm	Palliative	250	Nbib2721732

NCT, ClinicalTrials.gov registry number; RF, Radiofrequency.

**Table 4:** Guidelines on adrenal tumors.

Topic	Year	Organization	Reference
**Published during 2017**			
Adrenal incidentalomas, diagnosis	2017	ACR	Mayo-Smith *et al*. (2017)
PPGL, surveillance recommendations	2017	Consensus Committee	Rednam *et al*. (2017)
PPGL, genetic diagnosis and NGS	2017	Consensus Committee	Toledo *et al*. (2017)
ACC, surgery	2017	ESES & ENSAT	Gaujoux & Mihai (2017)
**Published earlier than 2017**			
Adrenal incidentalomas, diagnosis	2016	ENSAT	Fassnacht *et al*. (2016)
PPGL, management	2014	Endocrine Society	Lenders *et al*. (2014)
PPGL, follow-up	2016	ESE	Plouin *et al*. (2016)
Primary aldosteronism, management	2016	Endocrine Society	Funder *et al*. (2016)
Cushing syndrome, diagnosis	2008	Endocrine Society	Nieman *et al*. (2008)
Cushing syndrome, treatment	2015	Endocrine Society	Nieman *et al*. (2015)

ACC, adrenocortical carcinoma; ACR, American College of Radiology; ENSAT, European Network for the Study of Adrenal Tumors; ESE, European Society of Endocrinology; ESES, European Society of Endocrine Surgeons; NGS, next-generation sequencing; PPGL, pheochromocytoma and paraganglioma.

## Development and molecular characterization of the adrenal gland

Del Valle *et al.* studied adrenogonadal development during weeks 6–10 and characterized the processes of testis determination, onset of steroidogenesis and primordial germ cell development. Their genomic atlas of human adrenal and gonad development proposed new candidate genes for adrenal and reproductive disorders (Del Valle *et al*. 2017). The adrenal medulla is thought to originate from cells of the neural crest. Furlan *et al.* provided further evidence that multipotent peripheral glial cells also generate neuroendocrine cells of the adrenal medulla (Furlan *et al*. 2017).

The Human Protein Atlas (https://www.proteinatlas.org) aims to map all the human proteins in cells, tissues and organs using integration of various omics technologies. It has employed more than 26,000 antibodies on multiple tissues, cells and pathological states. The consortium now presented their data on the adrenal gland with RNA sequencing of tissue homogenates that identified 253 genes with an elevated expression pattern compared to other tissues (Bergman *et al*. 2017). Spatial expression patterns of the translated proteins were studied using immunohistochemistry.

## Incidentally discovered adrenal tumors

Clinical practice guidelines issued by the European Society of Endocrinology and ENSAT provide 19 recommendations, 16 of which are based on very low-quality evidence and 3 on low-quality evidence. The recommendations assert that adrenal tumors <4 cm with imaging characteristics of ≤10 Hounsfield units do not require further follow-up imaging. Recently, these data were now confirmed by a retrospective follow-up study (Hong *et al*. 2017). Conflicting data state that, for such patients, one-time follow-up evaluation involving a noncontrast CT and biochemical evaluation is cost-effective (Chomsky-Higgins *et al*. 2018).

For intermediate lesions, there is an increasing amount of data that ^18^F-fluorodeoxyglucose (FDG) PET/CT could improve diagnosis (Altinmakas *et al*. 2017, Delivanis *et al*. 2018). A prospective study showed that ^18^F-FDG PET/CT complements adrenal washout CT in the evaluation of adrenal masses with 86.7% sensitivity and 86.1% specificity for the detection of adrenocortical carcinoma (ACC) (Guerin *et al*. 2017). Finally, computational analysis of images data represents another venue to improve classification of adrenal tumors; Chai *et al*. performed a retrospective experiment where they reached a 90% accuracy analyzing 436 CT scans (Chai *et al*. 2017). Although prospective studies are lacking, we expect this field to evolve fast.

## PCC and PGL

**Figure 1:**
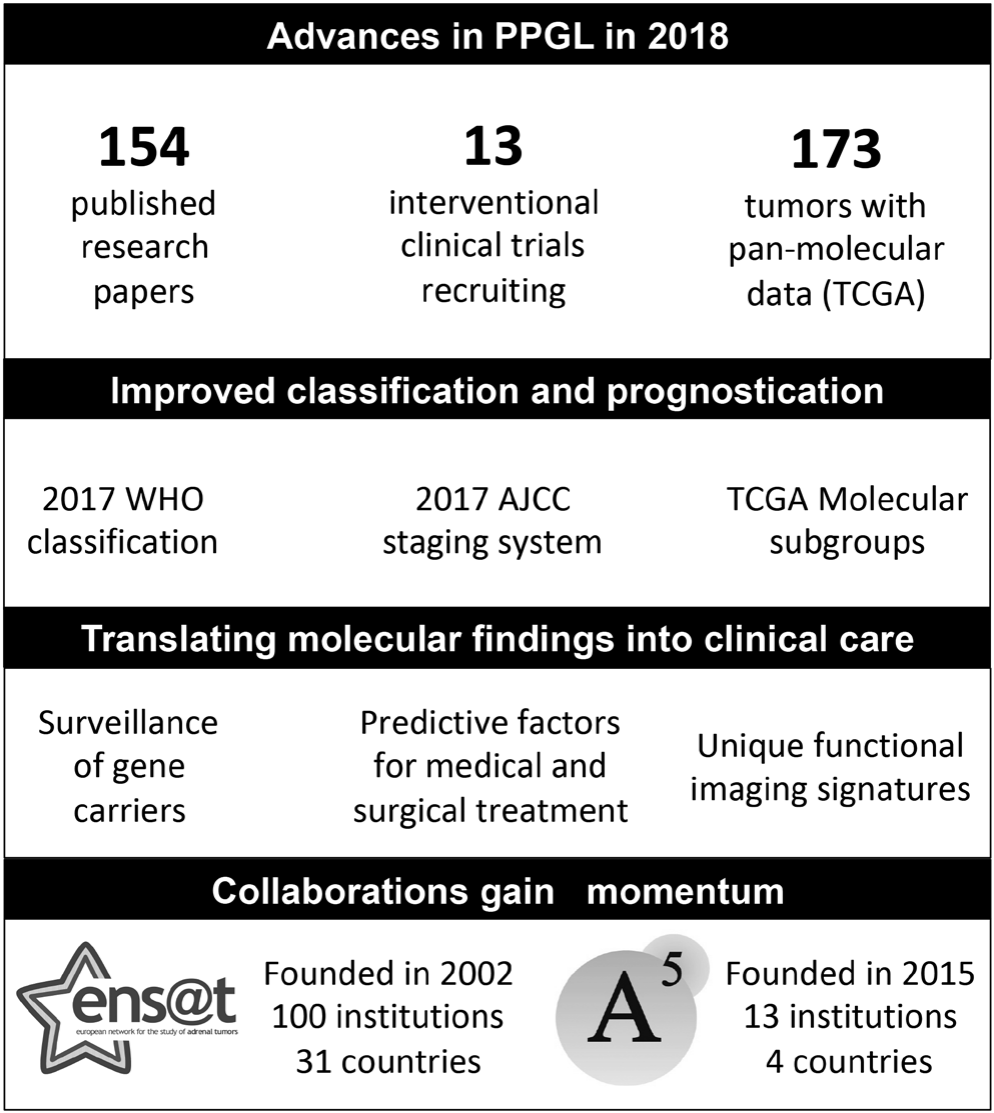
Advances in PPGL in 2018. A5, American-Australian-Asian Adrenal Alliance; AJCC, American Joint Committee on Cancer; ENSAT, European Network for the Study of Adrenal Tumors; PPGL, pheochromocytoma and paraganglioma; TCGA, The Cancer Genome Atlas; WHO, World Health Organization.

Our synthesis of the advances in PPGL is provided in Fig. 1.

## Clinical studies

### Classifications

The World Health Organization has released updated classifications of tumors from endocrine organs as well as the head and neck region. PPGLs are now classified as PCCs, sympathetic PGLs or head and neck (parasympathetic) PGLs (Lam 2017, Lloyd *et al*. 2017, Williams & Tischler 2017). Both classifications highlight the hereditary nature of PPGLs and emphasize that they ‘remain tumors of undetermined biologic potential and should not be termed benign’. This classification has been complemented by the first TNM staging system for PPGL released by the American Joint Committee on Cancer (Jimenez *et al*. 2017, Roman-Gonzalez & Jimenez 2017).

### Disease outcomes

#### Outcome from catecholamine excess

A study using the Nationwide Inpatient Sample studied outcomes after unilateral adrenalectomy among patient with hormonally active tumors (n = 6033), those with PCCs had a higher rate of comorbidities including congestive heart failure, chronic lung disease and malignant hypertension (Parikh *et al*. 2017). A systematic review identified 163 occurrences of PCCs and cardiomyopathy (Zhang *et al*. 2017). Hypertension was the presenting symptom in 65% and the classic triad of headache, palpitations and diaphoresis was observed in only 4%. PCC resection led to improvement of cardiomyopathy in 96% of patients, while lack of resection was associated with death or cardiac transplantation in 44% patients. Majtan* et al.* performed a prospective study on the effects of surgical resection on carotid intima-media thickness and left ventricular mass in 50 PPGL patients (Majtan *et al*. 2017). Both parameters significantly regressed after tumor removal; in contrast to patients with essential hypertension.

#### Outcome from metastatic disease

A meta-analysis on outcomes of patients with metastatic PPGLs showed a 5-year survival rate of 63% (95% CI, 49–76%) with poor survival associated with male sex and synchronous metastases (Hamidi *et al*. 2017*a*). The authors also describe the natural course of metastatic PPGLs at their referral center (Hamidi *et al*. 2017*b*). Among 272 patients, the median overall survival was 24.6 years. On the other hand, a subset of patients had a very aggressive disease course of the disease. This remarkably variability in patients affected by metastatic PPGL is supported by the authors’ own experience.

### Prospective clinical trials

#### High-specific activity I-131 meta-iodobenzylguanidine

In a phase 1 study, safety and efficacy of this compound was investigated in 21 metastatic PPGLs (Noto *et al*. 2018). The maximum tolerated dose was determined and the majority (84%) of adverse events were considered mild or moderate in severity. A 19% response rate on anatomical imaging was reported, only patients who received >18.5 GBq of study drug had a response.

#### Pazopanib

This phase II trial for advanced/progressive metastatic PPGL was halted due to poor accrual (Jasim *et al*. 2017). One of six patients had a partial response (duration 2.4 years). The authors stress that optimal alpha- and beta-adrenoceptor blockade is important in patients with secretory tumors to avoid risk of potentially life-threatening complications.

### Retrospective studies

#### Surgical treatment

In 53 PPGLs with synchronous metastases, patients with surgical resection of the primary tumor have a longer overall survival than those who did not (85 months vs 36 months) (Roman-Gonzalez *et al*. 2017). A second study investigated perioperative complications in 110 patients with and 166 without alpha-adrenoceptor blockade (Groeben *et al*. 2017). There was no difference in the incidence of excessive hypertensive episodes between the groups and no major complications occurred. The authors concluded that ‘the basis for the general recommendation of perioperative α-receptor blockade for PCC surgery demands further study’. A commentary stated that ‘when considering abandoning this conventional therapy altogether, one needs to carefully consider the confidence regarding the safety of this revised approach’ (Grocott 2017). In addition, this study does not consider preoperative safety that is of concern in patients with catecholamine-secreting tumors.

#### Oncological treatment

Efficacy and safety of peptide receptor radionuclide therapy (PRRT) (Kong *et al*. 2017), in combination with ^131^I-meta-iodobenzylguanidine (MIBG) (Nastos *et al*. 2017), interferon-alpha (Hadoux *et al*. 2017), and cyclophosphamide, vincristine and dacarbazine (CVD) were reported from retrospective materials. *SDHB* patients had a higher response rate to CVD compared to those without *SDHB* mutations (Asai *et al*. 2017, Fishbein *et al*. 2017*a*).

## Translating molecular findings to the clinic

### Disease penetrance and surveillance of gene carriers

Mutations in succinate dehydrogenase subunits A–D (*SDHx)* were thought to have almost complete penetrance for PPGL. This figure has now been adjusted, a finding of major importance when designing surveillance protocols for healthy carriers: the overall penetrance of *SDHB* mutations was estimated to be 21% at the age of 50 years and 42% at the age of 70 years (Rijken *et al*. 2018). A second study showed a penetrance of 49.8% at 85 years (Jochmanova *et al*. 2017). Penetrance of any *SDHA*-related manifestation in non-index patients was 13% at age 40 years (Bausch *et al*. 2017) and 10% at the age of 70 years (van der Tuin *et al*. 2018). The clinical spectrum associated with hereditary leiomyomatosis and renal cell carcinoma syndrome (associated with *FH* mutations) was assessed in 182 cases from 114 families and found only two cases of PPGL (Muller *et al*. 2017).

These findings align with a calculation of the optimal surveillance for head and neck PGL in *SDHB* mutation carriers; it has been suggested to start at the age of 27 with an interval of 3.2 years (Eijkelenkamp *et al*. 2017). The second study reported outcomes of annual surveillance imaging in *SDHB* mutation carriers (Tufton *et al*. 2017): in 27 index patients, 51 PPGLs (five metachronous) were detected. *SDHB*-related tumors occurred in 25% of asymptomatic carriers on surveillance screening: ten on the first surveillance imaging and five on subsequent imaging 2–6 years later. The authors suggested the use of annual or biannual imaging with MRI. These intervals are in line with recommendations on surveillance in childhood for some hereditary PPGLs (Rednam *et al*. 2017).

### Evaluation and treatment

#### Functional imaging

It is now established that the most optimal tracer for PET imaging can be selected from an underlying driver mutation. This knowledge was further refined in patients with polycythemia/PGL syndrome, optimal tracers being ^18^F-dihydroxyphenylalanine and ^18^F-fluorodopamine as well as pediatric *SDHx-*associated PPGL, optimal tracer being ^68^Ga-tetraazacyclododecane tetraacetic acid–octreotate (DOTATATE) (Janssen *et al*. 2017, Jha *et al*. 2017). Previous results also showed superiority of ^68^Ga-DOTATATE for detection of *SDHB*-related metastatic PPGL as well as PGLs of the head and neck (Janssen *et al*. 2015, Janssen *et al*. 2016). We believe that these findings will lead to a change in golden standard for staging and localization of PPGL from ^18^F-FDG-PET to a precision medicine-based approach. The norepinephrine transporter has a long-standing tradition as a target for ^123^I-MIBG scintigraphy, that has inborn limitations related to the imaging technology. Pandit-Taskar *et al.* described a PET analog, ^18^F-meta-fluorobenzylguanidine, that was safe, had favorable biodistribution and with good targeting of tumors (Pandit-Taskar *et al*. 2018).

#### Surgery

Preoperative genetic testing of PPGLs was found to influence the surgical approach and the extent of adrenal surgery (Nockel *et al*. 2018). Those with *RET, VHL* or *NF1* germline mutations more often had minimally invasive surgery with cortical-sparing adrenalectomy, whereas seven out of eight (87.5%) patients with *SDHB* mutation had an open approach. The underlying argument was to preserve cortical function in patients with a high risk of bilateral PCC having a low risk of metastatic disease (*RET* and *VHL* carriers), whereas those with a low risk of bilateral PPC but with risk factors for metastases (including *SDHB* and large tumor size) could benefit from an open approach to maximize probability of radical resection. A second study show that patients with NF type 1 had more volatile intraoperative course and more severe complications, probably related to larger tumors and abundant catecholamine secretion that resulted in a high proportion of open resections (Butz *et al*. 2017). 

#### Pathology

Koh *et al.* evaluated grading systems for predicting metastatic potential in PPGLs: they validated the grading system for PPGL (GAPP) and proposed a modified GAPP with addition of *SDHB* staining to be useful for the prediction of the metastatic potential and prognosis in PPGL (Koh *et al*. 2017).

## Translational research and basic science

### Molecular atlas of PPGL: the Cancer Genome Atlas study

The Cancer Genome Atlas described results from 173 PPGLs that were analyzed with six different molecular profiling technologies, the most comprehensive molecular characterization of PPGL ever performed (Fishbein *et al*. 2017*b*). All data are freely available online (https://gdc.cancer.gov) and serves to accelerate research in this rare entity. Disease-causing mutations or gene fusions occurred in 73% of cases. At least two novel disease-causing genes were identified: *MAML3* and *CSDE1*. Integrated analysis classified PPGL into three main subtypes: kinase signaling, pseudohypoxia and Wnt altered. A forth subtype, cortical admixture, was also detected but is thought to reflect a signal from non-tumoral cells.

Data from The Cancer Genome Atlas (TCGA) have already allowed PPGL to be included into several pan-cancer analyses; the first underscore that PPGL genomes exhibit relatively low number of somatic mutations as well as copy number segmentations compared to other tumors (Cancer Genome Atlas Network 2017). The second study investigated patterns of selection in cancer and confirmed known PPGL drivers as subjected to positive selection (Martincorena *et al*. 2017). Smaller studies complement the TCGA effort by demonstrating activating *FGFR1* mutations (Welander *et al*. 2018) as well as different landscapes of aneuploidy in *SDHB* versus *SDHAF2*, *SDHD* and *VHL*-related cases (Hoekstra *et al*. 2017). One aspect that TCGA did not cover was tumor evolution. Flynn *et al.* reported data from sequencing of tumors from syndromic patients with multiple PPGLs, different tumors shared rare somatic copy number suggesting that these changes could have been acquired early within common precursor cells (Flynn *et al*. 2017).

A third pan-cancer study performed a systematic analysis of telomere length, PPGLs were found to be exceptional as it lacks both telomerase reverse transcriptase (TERT) expression and mechanisms of alternative lengthening of telomeres in the highest proportion of cases (Barthel *et al*. 2017). Two mechanisms underlying increased TERT expression were identified: *TERT* structural rearrangements (Dwight *et al*. 2018) and *TERT* promoter hypermethylation (Svahn *et al*. 2018). These molecular data have allowed the stratification of PPGLs into subgroups have distinct molecular–biochemical–imaging signatures (reviewed in Crona *et al*. 2017).

### New treatment candidates and tumor models

Lack of representative tumor models has impeded progress toward new therapeutics for PPGLs. This year, patient-derived xenografts in NOD SCID gamma mice (Powers *et al*. 2017) as well as *SDHB*-silenced mouse PCC spheroids (D’Antongiovanni *et al*. 2017) were described and could potentially fill this gap. Effect of the following agents was suggested: HSP90 inhibitor (NVP-AUY922) in PC12 cells (Lian *et al*. 2017), anthracyclines through inhibition of the hypoxia signaling pathway in mouse PCC cell lines (Pang *et al*. 2017) and proteasome inhibitor (Bortezomib) in mouse PCC cell lines (Bullova *et al*. 2017). Rapalogues is approved for treatment for gastrointestinal neuroendocrine tumors but its efficacy in PPGLs has been doubted. This may have to be re-challenged as the mTORC1 complex was found to be overactivated in PPGL both of the head and neck and those harboring *SDHx* mutations (Oudijk *et al*. 2017).

## Adrenocortical tumors

**Figure 2:**
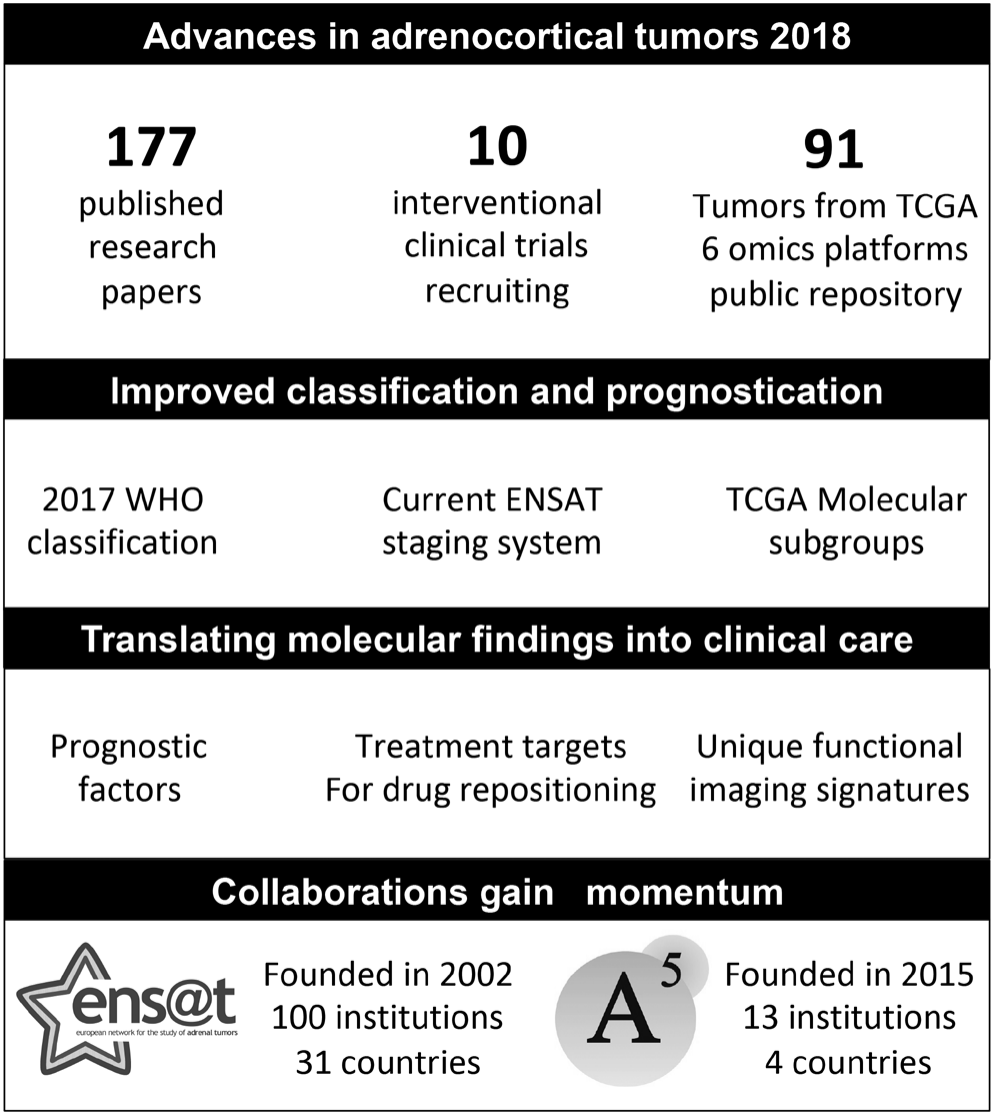
Advances in adrenocortical tumors in 2018. A5, American-Australian-Asian Adrenal Alliance; ENSAT, European Network for the Study of Adrenal Tumors; TCGA, The Cancer Genome Atlas; WHO, World Health Organization.

Our synthesis of the advances in adrenocortical tumors is provided in Fig. 2.

### Improved diagnosis

The 2017 World Health Organization classification recognizes Weiss score as the primary determinant of malignancy of adrenocortical tumors (Lam 2017, Lloyd *et al*. 2017). Three rare subtypes of ACC are recognized – oncocytic, myxoid and sarcomatoid carcinomas. Clinical and biochemical features together with absence of malignant criteria are used to diagnose adrenocortical adenoma (ACA). Thus, improved methods for subclassification of adrenocortical tumors are sought for: in three independent studies, the authors showed that different adrenal disorders have unique metabolic fingerprints that can be detected in serum or urine through mass spectrometry (Hines *et al*. 2017, Patel *et al*. 2017, Taylor *et al*. 2017). The aldosterone-to-renin ratio was evaluated prospectively for detection of aldosterone-producing adenoma (APA); baseline and post-captopril aldosterone-renin ratio showed similar diagnostic accuracy in both the exploratory and validation cohorts, indicating lack of diagnostic gain with this confirmatory test (Maiolino *et al*. 2017). Radiographically identified adrenal nodules are not always a source of primary aldosteronism (PA), even when ipsilateral lateralization on adrenal vein sampling occurs (Nanba *et al*. 2017). One alternative could be targeting CXC chemokine receptor type 4 for functional imaging classification of both APA (Heinze *et al*. 2018) and ACC (Wu *et al*. 2017).

## Adrenocortical adenoma

### Outcomes

A prospective study determined the prevalence of PA among 1672 primary care patients with hypertension: 5.9% had PA and one-third of these had a unilateral APA accordingly to adrenal CT scanning and adrenal vein sampling (Monticone *et al*. 2017). A meta-analysis of 3838 patients with PA revealed an increased risk of stroke (OR 2.58, 95% CI 1.93–3.45), coronary artery disease (1.77, 1.10–2.83), atrial fibrillation (3.52, 2.06–5.99) and heart failure (2.05, 1.11–3.78) compared to patients with essential hypertension (Monticone *et al*. 2018). Patients with PA were also shown to have deteriorated bone quality without loss of overall bone mass (Kim *et al*. 2018).

Data from the randomized SPARTACUS trial was used to study quality of life in PA patients: 1 year post adrenalectomy, quality of life normalized, whereas for patients on medical treatment, quality of life had improved but was inferior to the level of the general population (Velema *et al*. 2018).

### Cortisol secretion: utility in multiple scenarios

Arlt *et al.* analyzed steroid metabolome in 174 newly diagnosed patients with PA (103 APA, 71 bilateral adrenal hyperplasia) (Arlt *et al*. 2017). Patients with PA had significantly increased cortisol and total glucocorticoid metabolite excretion, only exceeded by glucocorticoid output in patients with clinically overt adrenal Cushing syndrome. In a second series, 4/5 APAs with concurrent subclinical cortisol hypersecretion were found to be composed of zona fasciculata-like cells, with heterogenous CYP11B1 and CYP11B2 immunostaining and lacking driver mutations associated with APA (Fallo *et al*. 2017).

Two studies confirmed the association between non-functional ACA with autonomous cortisol secretion to cardiovascular disease (Arruda *et al*. 2017) as well as increased mortality (Patrova *et al*. 2017). This was corroborated in a third study that suggested increased visceral fat as an explanation (Yener *et al*. 2017).

Finally, perioperative ACTH, steroid precursors and tumor size was found to predict recurrence of Cushing’s disease (El Asmar *et al*. 2018).

## Translating molecular findings to the clinic: new treatment options

Molecular characterization of APA has pinpointed key disease driving mechanisms that are now exploited by researchers as biomarkers and therapeutic targets (reviewed in Zennaro *et al*. 2017).

### Surgical therapy

Kitamoto *et al.* correlated outcome to somatic mutation status among 142 patients with APA;* KCNJ5* mutations in young patients with APA emerged as a prognostic biomarker indicating resolution of hypertension (Kitamoto *et al*. 2018). A second study found that *CTNNB1*-mutated APA had a higher possibility of residual hypertension (Wu *et al*. 2017).

### Medical therapy

Following the discovery of somatic mutations in *KCNJ5* (potassium channel) as a driver of APA, macrolides were shown to selectively inhibit mutant *KCNJ5* opening, which might provide the option for improved diagnosis and treatment (Caroccia *et al*. 2017, Scholl *et al*. 2017). Other potential therapeutic targets of PA included the E3 ubiquitin ligase Siah1 (Scortegagna *et al*. 2017), neurofilament medium polypeptide (Maniero *et al*. 2017) and calneuron 1 (Kobuke *et al*. 2018).

## Translational research and basic science

### APA

Biology and clinicopathological characteristics of this disorder are dependent on the mutational status, an expanding field that came up with several clarifying publications (Murakami *et al*. 2017, Tan *et al*. 2017). However, whether PA is the consequence of a monoclonal or multiclonal processes is still not clear. Aging was found to correlate with remodeling of the adrenal cortex and emergence of subcapsular aldosterone-producing cell clusters (APCCs) that replaced the continuous zona glomerulosa layer. In a first study, the authors provided evidence that PA involves polyclonal APAs (Omata *et al*. 2017*b*). A second study studied 107 unilateral adrenal glands obtained from autopsies of nonhypertensive patients. Sixty-one APCCs were detected (average of 0.6 APCCs per gland) (Omata *et al*. 2017*a*). In a third study of PA patients with negative cross-sectional imaging, the resected adrenal gland showed that 13 had multiple adrenocortical micronodules and 12 had diffuse hyperplasia of zona glomerulosa based upon histopathological evaluation and CYP11B2 IHC. Aldosterone-driver gene somatic mutations were detected in 21 of 26 (81%) of CYP11B2-positive cortical micronodules (Yamazaki *et al*. 2017). Finally, a study reported six patients with possible APCC-to-APA transitional lesions (Nishimoto *et al*. 2017). These data questions if the current classification that recognizes either unilateral APA or bilateral hyperplasia is relevant especially as future personalized treatment options might be based on molecular findings rather than tumor size.

### Cortisol-producing adenoma

Protein kinase A catalytic alpha subunit is a disease driver in 30–40% of cortisol-producing adenoma (CPA) and was associated with reduced DNA methylation at the CYP11B1 promoter that may result in CYP11B1 transcription and hypercortisolemia (Kometani *et al*. 2017). A second study characterized expression of the protein kinase A subunits in normal adrenal glands and ACA (Weigand *et al*. 2017). The molecular etiology behind a rare subtype of Cushing syndrome caused by ectopic expression of glucose-dependent insulinotropic polypeptide receptor (GIPR) was unveiled; microduplications at chromosome 19q13 that contain the *GIPR* locus (Lecoq *et al*. 2017).

## Adrenocortical carcinoma

### Outcome and prognostic factors

Partial response (PR) has been proposed as a surrogate for overall survival in ACC. This study found that most patients with metastatic ACC and long survival times had PR within the first 6 months of systemic therapy, and almost all within the first year. The absence of response after that period could be considered as a treatment failure (Vezzosi *et al*. 2018). Eighty-two patients with high-risk pediatric ACC were evaluated for outcome and prognostic factors: distant metastases and large tumor volume were associated with unfavorable prognosis (Cecchetto *et al*. 2017).

### Improved classification and prognosis

It is clear that the most optimal clinicopathological classification of ACC has yet to be determined. The United States ACC Study Group analyzed 149 patients and proposed a refined TNM classification with a novel T-Stage (Poorman *et al*. 2018). In a second study, the Helsinki Score, a diagnostic and prognostic system based on the combined evaluation of mitoses and necrosis as well as Ki-67 index, was investigated in 225 cases of ACC (Duregon *et al*. 2017). The third study found that low mitotic tumor grade, Weiss score, global loss of DAXX expression and high phospho-mTOR expression correlated with disease-free survival (Mete *et al*. 2018).

### Clinical studies

#### Prospective clinical trial

A Phase 1 study of ARQ 087, an oral pan-FGFR inhibitor, was investigated in patients with advanced solid tumors (Papadopoulos *et al*. 2017). This was a basket trial that included one patient with a *FGFR1*-amplified ACC that showed stabled disease upon treatment.

#### Retrospective studies, surgical treatment

Outcome after resection of ACC liver metastases was studied in 77 patients without extrahepatic disease (Baur *et al*. 2017). The median overall survival was 76.1 months in 43 patients that underwent metastasectomy, compared to 10.1 months in the 34 patients without surgical resection. However, the median disease-free survival in resected ACC was only 9.1 months. A second study investigated perioperative blood transfusion that has been associated with decreased survival in pancreatic, gastric and liver cancer. Perioperative transfusion was associated with earlier recurrence and decreased survival after curative-intent resection of ACC (Poorman *et al*. 2017). Another study proposed a threshold for surgeon volume to minimize complications and decrease cost associated with adrenalectomy (Anderson *et al*. 2018). A total of 3496 surgeons performed adrenalectomies on 6712 patients; median annual surgeon volume was 1 case. After adjustment, the likelihood of experiencing a complication decreased with increasing annual surgeon volume up to 5.6 cases (95% CI, 3.27–5.96). Recommendations for the perioperative surgical care of patients with ACC from the European Society of Endocrine Surgeons and ENSAT are now available (Gaujoux & Mihai 2017).

#### Oncological treatment

One hundred forty-five ACC received gemcitabine based chemotherapy, PR or stable disease was achieved in 4.9 and 25.0%, respectively (Henning *et al*. 2017). No predictive factors could be identified. Claps *et al.* reported that the combination of metyrapone with mitotane, etoposide, doxorubicin and cisplatinin (EDP-M) achieved rapid control of Cushing syndrome induced by cortisol-secreting ACC in three patients (Claps *et al*. 2017). *ERCC1*, involved in DNA excision repair, was investigated as a predictive biomarker of platinum-based chemotherapy in 146 ACC but demonstrated negative results (Laufs *et al*. 2018). A second predictive marker topoisomerase II alpha, showed a positive correlation to EDP-M, disease response or stabilization was observed in 21/30 topoisomerase II alpha positive ACC compared to 5/22 in those without the biomarker (Roca *et al*. 2017).

## Translating molecular findings to the clinic

Comprehensive characterization of ACC biology was previously achieved by TCGA and ENSAT consortiums that together proposed a robust molecular classification (Assie *et al*. 2014, Zheng *et al*. 2016). Prognostic impacts of these subgroups were analyzed using methodologies compatible with clinical diagnostic use. Jouinot *et al.* investigated DNA methylation and Sbiera *et al.* investigated *VAV2* gene expression (Jouinot *et al*. 2017, Sbiera *et al*. 2017). Both methods revealed a strong correlation to survival that was independent to traditional measures in their multivariate analyses. These studies will pave the way of including new prognostic biomarkers into the traditional classification of ACC.

A study of 60 pediatric ACCs investigated the impact of germline *TP53* mutations and showed similar prognosis and outcome regardless of mutation status. Ki67 index was a promising prognostic biomarker also in pediatric ACC (Pinto *et al*. 2017).

### Liquid biopsy

Independent studies show that circulating tumor DNA can be found in a subset of ACC with high tumor burden (Creemers *et al*. 2017, Garinet *et al*. 2018). These early data implies that liquid biopsy has the potential to be used to estimate relative changes in tumor volume as well as to determine the genetic composition of a subset of ACC. Traces of extracellular vesicle-associated microRNAs in the blood was also found to be useful but for a different purpose; perioperative diagnosis of ACC (Perge *et al*. 2017).

## Translational research and basic science

### New genetic risk factors

Five out of 21 patients with *MUTYH*-associated polyposis had adrenal lesions; two were hyperfunctioning. Among four patients that underwent adrenalectomy, three had benign tumors and one was oncocytic of uncertain malignant potential (Kallenberg *et al*. 2017). Traces of MUTYH deficiency can be found in tumor mutatomes through a unique signature of DNA mutations. Pilati *et al.* were able to detect this MUTYH deficiency signature in ACC (Pilati *et al*. 2017). Finally, succinate dehydrogenase gene mutations were found in four unrelated patients with cortisol-secreting ACC (Else *et al*. 2017). A majority lacked molecular hallmarks associated to *SDHx* deficiency.

### ACC pathogenesis

The TCGA dataset has now allowed researchers not involved in the adrenal field to characterize ACC and compare it to other cancers. Three different studies compared the mutational landscape (Cancer Genome Atlas Network 2017), patterns of selection (disease evolution) (Martincorena *et al*. 2017) as well as telomere length and somatic alterations (Barthel *et al*. 2017).

### New treatment candidates and tumor models

#### CDK4-6 inhibitors

CDK4 and CDK6 inhibitors were suggested to be candidate drugs for treatments of ACC (Hadjadj *et al*. 2017) and a second study identified palbociclib to inhibit proliferation of human adrenocortical tumor cells (Fiorentini *et al*. 2018).

#### Aurora kinase inhibitors

The aurora kinase inhibitor AMG 900 increased apoptosis and chemosensitivity to anticancer drugs in the NCI-ACC cell line (Borges *et al*. 2017).

#### Guanine nucleotide exchange factor VAV2

VAV2 was mentioned earlier as a prognostic factor, this study revealed molecular mechanisms involved and suggest that blocking VAV2 may be a new therapeutic approach to inhibit metastatic progression (Ruggiero *et al*. 2017).

#### Rotterin

Rottlerin was introduced as a novel chemotherapy agent (Zhu *et al*. 2017) and synthetic high-density lipoprotein nanodisks for targeted delivery to ACC (Kuai *et al*. 2017).

#### mTOR and SSTR2 pathways

Analyses of the mTOR and SSTR2 pathways in ACC cell lines H295R and SW13 (Germano *et al*. 2017) revealed that everolimus monotherapy and combinations with either mitotane or pasireotide resulted in growth inhibition.

#### Acyl-CoA acyltransferase 1 inhibition

ATR-101 was found to inhibit cholesterol efflux and cortisol secretion by ATP-binding cassette transporters, causing cytotoxic cholesterol accumulation in ACC (Burns & Kerppola 2017). This compound is of high interest in the treatment of adrenocortical tumors including ACC.

#### Tumor models

A mouse xenograft model of metastatic ACC (Morin *et al*. 2017) and a transgenic mouse model of metastatic ACC through P53/Rb inhibition (Batisse-Lignier *et al*. 2017) were described.

## Conclusions

Clinical and basic research on adrenal tumors is an active field that generated very promising advances during 2017. Prominent examples include an improved understanding of adrenal tumor molecular pathogenesis as well as the introduction of new classifications, molecular markers and tracers for functional imaging. We also highlight international collaboration as a key factor that is likely to accelerate improvements in treatment and outcome of patients with these tumors.

## Declaration of interest

J C received lecture honoraria from Novartis.

## Funding

J Cs research position is funded by Akademiska Sjukhuset, Uppsala, Sweden.
